# Peripheral metabolic alterations associated with pathological manifestations of Parkinson’s disease in gut-brain axis-based mouse model

**DOI:** 10.3389/fnmol.2023.1201073

**Published:** 2023-08-10

**Authors:** Eugene Huh, Jin Gyu Choi, Mee Youn Lee, Jin Hee Kim, Yujin Choi, In Gyoung Ju, Hyeyoon Eo, Myoung Gyu Park, Dong-Hyun Kim, Hi-Joon Park, Choong Hwan Lee, Myung Sook Oh

**Affiliations:** ^1^Department of Oriental Pharmaceutical Science and Kyung Hee East-West Pharmaceutical Research Institute, College of Pharmacy, Kyung Hee University, Seoul, Republic of Korea; ^2^Department of Bioscience and Biotechnology, Konkuk University, Seoul, Republic of Korea; ^3^Department of Biomedical and Pharmaceutical Sciences, Graduate School, Kyung Hee University, Seoul, Republic of Korea; ^4^MetaCen Therapeutics Inc. R&D Center, Suwon, Republic of Korea; ^5^Neurobiota Research Center, College of Pharmacy, Kyung Hee University, Seoul, Republic of Korea; ^6^Acupuncture and Meridian Science Research Center (AMSRC), College of Korean Medicine, Kyung Hee University, Seoul, Republic of Korea; ^7^Department of Integrated Drug Development and Natural Products, Graduate School, Kyung Hee University, Seoul, Republic of Korea

**Keywords:** Parkinson’s disease, microbiota-gut-brain axis, endogenous metabolites, *Proteus mirabilis*, glycation

## Abstract

**Introduction:**

Parkinson’s disease (PD) is a representative neurodegenerative disease, and its diagnosis relies on the evaluation of clinical manifestations or brain neuroimaging in the absence of a crucial noninvasive biomarker. Here, we used non-targeted metabolomics profiling to identify metabolic alterations in the colon and plasma samples of *Proteus mirabilis* (*P. mirabilis*)-treated mice, which is a possible animal model for investigating the microbiota-gut-brain axis.

**Methods:**

We performed gas chromatography–mass spectrometry to analyze the samples and detected metabolites that could reflect *P. mirabilis*-induced disease progression and pathology.

**Results and discussion:**

Pattern, correlation and pathway enrichment analyses showed significant alterations in sugar metabolism such as galactose metabolism and fructose and mannose metabolism, which are closely associated with energy metabolism and lipid metabolism. This study indicates possible metabolic factors for *P. mirabilis*-induced pathological progression and provides evidence of metabolic alterations associated with *P. mirabilis*-mediated pathology of brain neurodegeneration.

## Graphical Abstract

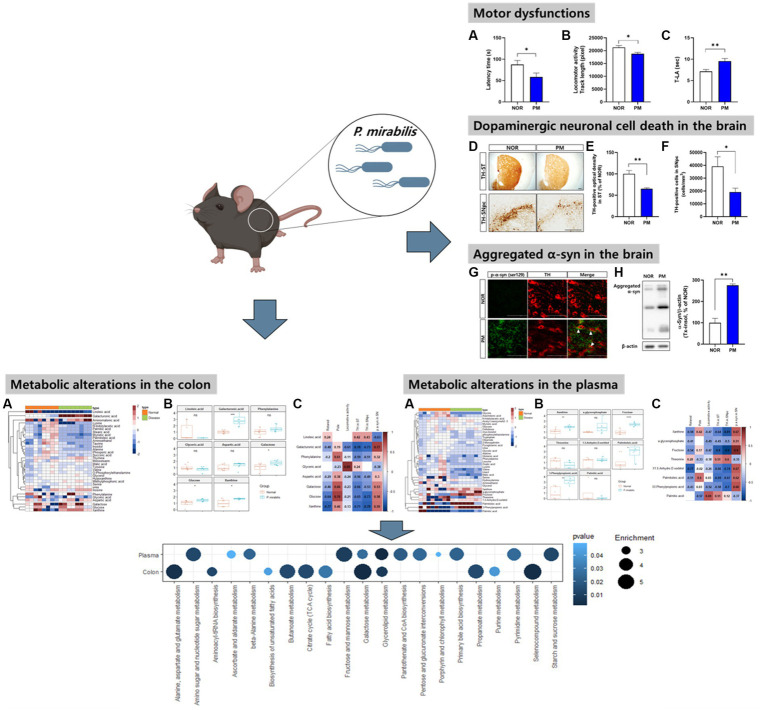

## Introduction

Parkinson’s disease (PD) is the second most common neurodegenerative disease affecting approximately 1% of the population over 60 years following Alzheimer’s disease. Its neuropathological characteristics include a marked loss of dopaminergic neurons within the substantia nigra and the presence of intracytoplasmic α-synuclein-containing Lewy bodies, manifesting as reduced facilitation of voluntary movement and increased rigidity (Espay et al., 2020). The current diagnosis of PD relies on the evaluation of clinical signs. Although neuroimaging technologies have improved the diagnosis and staging of PD modalities, these detections are expensive and labor intensive (Tremblay et al., 2020). Thus, various studies have been conducted to identify biomarkers that may assist in the diagnosis of PD.

To overcome the limitations of the current diagnosis of PD, a number of trials investigating metabolic alterations in peripheral regions (i.e., plasma, serum, sebum and feces) have been reported for PD biomarker (Hatano et al., 2016; Weng et al., 2019; Shao et al., 2021; Sinclair et al., 2021). Studies have revealed that the plasma levels of bilirubin and ergothioneine are related to a decrease in oxidative stress and suggest a potential biomarker to identify the metabolites involved in the pathological mechanism of PD (Hatano et al., 2016). Conversely, promising metabolites such as quinolinic acid/kynurenic acid ratio, N8-acetyl spermidine and polyunsaturated fatty acids have been revealed through continuous analyses (Schulte et al., 2016; Chang et al., 2018; Shao and Le, 2019). Recent studies have focused on the correlation between peripheral metabolic alterations and PD manifestations such as motor or non-motor symptoms, especially in the microbiota-gut-brain axis (MGB axis). Shao et al. found microbiota-derived deleterious metabolites such as *p*-cresol sulfate, *p*-cresol glucuronide, and phenylacetyl-_L_-glutamine, which were reported to be correlated with constipation, as indicated by changes in intestinal homeostasis and consequent non-motor symptoms of PD (Shao et al., 2021). Previous studies have established biomarker by analyzing the correlation between metabolites and brain factors or phenotypes in an *in vivo* PD model in progress (Graham et al., 2018); however, these are still insufficient.

Several studies have reported that changes in peripheral biological factors in PD are closely connected to the etiology and progression of the disease. Among them, the MGB axis is the concept that receives the most attention in explaining the crosstalk between the periphery and the brain. Numerous studies have reported changes in the microbial composition of PD patients (Weis et al., 2019; Baizabal-Carvallo and Alonso-Juarez, 2020; Cirstea et al., 2020). In addition, animal studies have demonstrated that the intestinal microbiota and its virulence factors could exacerbate PD progression, whereas well-defined metabolic alterations based on the MGB axis have not yet been elucidated. We previously reported that mice administered a specific bacterium orally, *Proteus mirabilis* (*P. mirabilis*) induced motor deficits. When treating *P. mirabilis*, we found that dopaminergic neuronal death, neuroinflammation and α-synuclein aggregation were induced in the brain, and α-synuclein aggregation and *P. mirabilis*-derived lipopolysaccharide were increased in the colon, simultaneously (Choi et al., 2018). Though we previously demonstrated the pathogenesis of *P. mirabilis*-induced brain neurodegeneration partly, it is crucial to investigate further studies such as metabolic analysis to reveal the clear pathological mechanism of *P. mirabilis*-induced neurological pathology.

Prior to the current study, it was confirmed that metabolites were altered in a different pattern when compared to the normal and 1-methyl-4-phenyl-1,2,3,6-tetrahydropyridine (MPTP)-induced PD mouse models (Supplementary Figure S1). Since the model we constructed has been regarded as a tool for investigating the MGB axis in PD, the metabolic alterations shown in this model might be helpful in understanding PD pathologies. Herein, we utilized a gas chromatography mass spectrometry (GC–MS)-based non-targeted metabolomics approach to investigate the metabolic changes associated with PD using *P. mirabilis*-treated mouse model based on the MGB axis. We also presented a comprehensive analysis of metabolic profiles using targeted extraction and integration of the chromatographic peak. Additionally, the relationship between various variables (behavioral and neuropathological manifestations) and metabolite levels was investigated. We aimed to identify the most critical metabolic biomarkers for neurodegenerative diseases, and the corresponding metabolic pathways that may contribute to a better understanding of the biochemical impairments involved in the disease.

## Materials and methods

### Materials

Paraformaldehyde (PFA), phosphate buffer, phosphate buffer saline, ethylene glycol, glycerol, sucrose, hydrogen peroxide, 3,3′-diaminobenzidine, methanol, methoxyamine hydrochloride, N-methyl-N-(trimethylsilyl) trifluoroacetamide, anti-tyrosine hydroxylase (TH) antibody (AB152), skim milk and polyvinylidene fluoride (PVDF) membrane were purchased from Merck Millipore (Burlington, MA, United States). Anti-α-syn antibody (610787) was purchased from BD biosciences (Franklin Lakes, NJ, United States). Goat anti-mouse HRP secondary antibody (ADI-SAB-100) was purchased from Enzo life sciences (Farmingdale, NY, United States). Goat anti-Mouse Secondary antibody (Alexa Fluor 488, A-11001) was purchased from Invitrogen (Waltham, MA, United States). Biotinylated goat anti-rabbit IgG antibody (BA-1000), avidin-biotin complex and Goat anti-Rabbit Secondary antibody (DyLight Fluor 594, DI-1594) were purchased from Vector Laboratories (Burlingame, CA, United States).

### *Proteus mirabilis* culture

*P. mirabilis* used in this study was isolated in the previous study. Isolated *P. mirabilis* was cultured in GAM broth at 37°C for 24 h under anaerobic condition and centrifuged for harvest.

### Animals and administration

Seven-week-old male C57BL/6 J mice used in this study were purchased from Daehan Biolink (Eumseong, Korea). After the adaptation for 7 days, mice were housed in separate cages per group (n = 8 per cage) at an ambient temperature of 23 ± 1°C and relative humidity 60 ± 10% under a 12 h light/dark cycle and were allowed free access to water and food. All animal studies were performed in accordance with the “Principles of Laboratory Animal Care” (NIH publication number 80–23, revised 1996) and approved by the “Animal Care and Use Guidelines” of Kyung Hee University, Seoul, Korea (approval number: KHUASP (SE)-20–029).

### Administration of *Proteus mirabilis*

Mice in the *P. mirabilis* group were treated with *P. mirabilis* and was described in the previous study (Choi et al., 2018). Briefly, *P. mirabilis* was orally administered to mice for 5 days (2 × 10^8^ CFU/0.2 mL PBS per mice). The normal group was orally administered equal volume of PBS for 5 days. Mice were sacrificed on the 16^th^ day after the last administration of *P. mirabilis*.

### Behavior tests

Behavior tests were conducted as previously described (Choi et al., 2018). Brief information was provided as follows.

#### Pole test

We performed a pole test on the 15th day after *P. mirabilis* administration. The mice were held on top of the pole (diameter, 8 mm; height, 55 cm; rough surface). The time required for the mice to climb down and place all four feet on the floor was recorded with a 30 s cut-off limit.

#### Open field test

The open field test was performed between 9 p.m. and 2 a.m. to avoid diurnal variations. The mice were placed in a testing chamber (40 × 25 × 18 cm) with white floors, followed by a 30-min recording period using a computerized automatic analysis system (Viewer; Biobserve, Bonn, Germany). The data collected by the computer included the total distance traveled by tracking the center of the animal.

#### Rotarod test

We performed a rotarod test 16 day after the last administration of *P. mirabilis*. The rotarod unit consists of a rotating spindle (7.3 cm diameter) and five individual compartments. After three trainings (8–10 rpm rotation speed), the rotation speed was increased to 16 rpm during the test session. The time that each mouse remained on the rotating bar was recorded over three trials per mouse, with a maximum length of 3 min per trial. The data are presented as the mean time of the rotating bar over three test trials.

### Biological sample preparation

For immunohistochemical studies, mice were perfused transcardially with 0.05 M PBS, and then fixed with cold 4% PFA in a 0.1 M phosphate buffer. Brains were removed and post-fixed in a 0.1 M phosphate buffer containing 4% PFA overnight at 4°C and then immersed in a solution containing 30% sucrose in 0.05 M phosphate buffer saline for cryoprotection. Serial 30 μm-thick coronal sections were cut on a freezing microtome (Leica, Germany) and stored in cryoprotectant (25% ethylene glycol, 25% glycerol, and 0.05 M phosphate buffer) at 4°C until use. For metabolomic analysis, the mice were decapitated and the distal colons and plasma were isolated and stored at −80°C until use.

### Immunohistochemistry

The floating brain sections of the striatum (ST) region (AP 0 to 0.5 mm) and substantia nigra pars compacta (SNpc) region (AP -3.5 to −3.0 mm) were incubated overnight with a rabbit anti-TH (1:1000) after reacting with 1% hydrogen peroxide. They were subsequently incubated with a biotinylated goat anti-rabbit IgG antibody (1:200), followed by incubation in an avidin-biotin complex solution. 3,3′-diaminobenzidine was used to develop the color of every section and the images were photographed using an optical light microscope (Olympus Microscope System BX51; Olympus, Tokyo, Japan). The optical density of TH-positive fibers was measured in the ST at × 40 magnification using Image J software (National Institutes of Health, Bethesda, MD, United States). The TH-positive cells in the SNpc were measured at × 200 magnification using Image J software. Optical density measurement and cell counts were determined by an experimenter who was blinded to the treatment conditions, and the outcome for each animal was the mean of the results from the three sections.

Stereological quantification for SNpc was performed as previously described (Sim et al., 2017). Briefly, the total number of TH-positive neuronal cells were counted in every A9 neurons of SNpc section for delimiting from the ventral tegmental area (A10). We also showed the data by the number of TH-positive cells per volume (mm^3^).

### Immunofluorescence

For immunofluorescence, brain tissues were rinsed in PBS and incubated overnight with rabbit anti-TH (1:1000) and mouse anti-phosphorylated α-syn (1:1000) antibodies. They were then incubated with goat anti-rabbit DyLight 594, goat anti-mouse Alexa 488 (1:500) at room temperature. Images were visualized using confocal microscopy at × 400 magnification [K1-Fluo; Nanoscope Systems (Daejeon, Korea)].

### Western blotting

To prepare protein for western blot for α-syn, brain tissues were lysed in PBS containing 10% Triton X-100 and protease/phosphatase inhibitor cocktail. After incubation on ice for 20 min, pellets were lysed in 50 mM Tris–Hcl containing 2% SDS and protease/phosphatase inhibitor cocktail (insoluble fraction). 20 μg of protein extract was separated on 12% polyacrylamide gel and transferred to a PVDF membrane. Membranes were blocked with 5% skim milk in TBS followed by incubated in 1% nonfat milk in TBS-T containing anti-α-syn (1:1000) and anti-β-actin (1:3000) antibodies overnight at 4°C. Membranes were washed in TBS-T and incubated in 1% skim milk in TBS-T containing goat anti-mouse HRP secondary antibody (1:3000) at room temperature. Membranes were imaged using Image Lab software (Bio-Rad, CA, United States).

### Sample preparation for metabolomics

The plasma samples were extracted by adding 1 mL of ice-cold 100% methanol to 120 μL of mouse plasma. Frozen colon (100 mg) were added to 1 mL of ice-cold 80% methanol. 4 samples per group were utilized. Each sample mixture was homogenized (frequency = 30 Hz) for 10 min using a Retsch MM400 mixer mill (Retsch GmbH & Co, Haan, Germany) and was kept at −20°C for 1 h. Then, the sample was centrifuged at 4°C and 12,578 g for 10 min. The supernatants were passed through a 0.2-μm polytetrafluoroethylene syringe filter and transferred to Eppendorf tubes. The supernatant was completely dried using a speed vacuum machine and stored in a −80°C. Dried extracts were reconstituted with 100% methanol to a final concentration of 10 mg/mL for instrumental analysis and syringe-filtered prior to the GC–MS analysis. The dissolved samples (100 μL) were dried again using a speed vacuum concentrator prior to the two-stage derivatization step.

For the GC–MS analysis, the dried sample was oximated with 50 μL of methoxyamine hydrochloride in pyridine (20 mg/mL) for 90 min at 30°C using a thermomixer (Eppendorf, Hamburg, Germany). Then, the oximated samples were silylated with 50 μL of N-methyl-N-(trimethylsilyl) trifluoroacetamide (MSTFA) for 30 min at 37°C using a thermomixer. Pooled quality control (QC) samples were prepared from 50 μL blends of each sample. Four biological (10,000 ppm each) and two analytical replicates for each of the extracted samples were analyzed using both GC-TOF-MS. The analytical samples were analyzed in blocks of seven runs followed by intermittent QC analysis to ensure the data quality and robustness of the method.

### GC-TOF-MS analysis

The conditions of GC-TOF-MS analysis were described based on a previous study (Ju et al., 2020). GC-TOF-MS analysis was performed using an Agilent 7890A GC system (Palo Alto, CA, United States) coupled with a Leco TOF Pegasus III mass spectrometer. Metabolites were separated using a Rtx-5MS column (30 m × 0.25 mm I.D. × 0.25 μm, J & W Scientific, Folsom, CA, United States) with helium as the carrier gas at a constant flow rate of 1.5 mL/min. A total of 1 μL of the derivatized sample was injected in the split mode (1:10). Before and after each injection, the 10 μL injection syringe was washed 3x with 10 μL of hexane and methanol. The oven temperature was maintained at 75°C for 2 min, then increased to 300°C at a rate of 15°C/min and held for 3 min. The mass data were collected in electron ionization mode with an ionization energy of 70 eV and mass scan (*m/z*) range of 50–600 at an acquisition rate of 20 spectra/s. The injector and ion source temperatures were set at 250 and 230°C, respectively.

### Data processing and statistical analysis

The GC-TOF-MS raw data processing and multivariate statistical analysis were conducted as described in our previous study (Ju et al., 2020). Raw data were converted to NetCDF format (*.cdf) using ChromaTOF (version 4.44, LECO). After conversion, MS data were processed using the Metalign software package^1^ to obtain a data matrix containing retention times, accurate masses and normalized peak intensities. The resulting data were exported to Excel (Microsoft, Redmond, WA, United States) for multivariate analysis. The aligned peaks were confirmed in the original chromatograms and were positively or tentatively identified using either commercial standard compounds in comparison with the mass spectra and retention time or on the basis of the NIST mass spectral database, in-house library, and references for GC-TOF–MS. Multivariate data analyses were performed using SIMCA-P+ software (version 12.0, Umetrics, Umea, Sweden). Principal component analysis (PCA) and partial least squares discrimination analysis (PLS-DA) were performed to compare the experimental groups. Significantly discriminant metabolites with variable importance in projection (VIP) value exceeding 0.7 was obtained using the orthogonal partial least square discriminant analysis (OPLS-DA) model. In OPLS-DA, the discriminated variables were selected based on variable importance in the projection value and checked with *value of p* from one-way ANOVA. The differential metabolites were subsequently identified by comparison of the obtained mass fragment patterns with those in the NIST library, Wiley 9th database, and of standard compounds. Multivariate receiver operating characteristic (ROC) curve-based exploratory analysis was executed using the MetaboAnalyst 5.0. Biomarker Analysis in which the data matrix was auto-scaled and OPLS-DA was used for the classification method, and feature ranking method with a two latent variable input.

### Pathway analysis

Pathway analysis was performed using the MetaboAnalyst 5.0. During the analysis a list of refined significant metabolites was generated using Student’s t-test (*p*-value <0.05). Significant features were mapped onto a combination of metabolic models: the Kyoto Encyclopedia of Genes and Genomes model. Feature hits on known metabolite networks were tested against a null distribution produced from permutations of features to yield significance values for metabolites enriched within any given network.

## Results

### Analysis of manifestations of *Proteus mirabilis*-treated mouse

**Figure 1:**
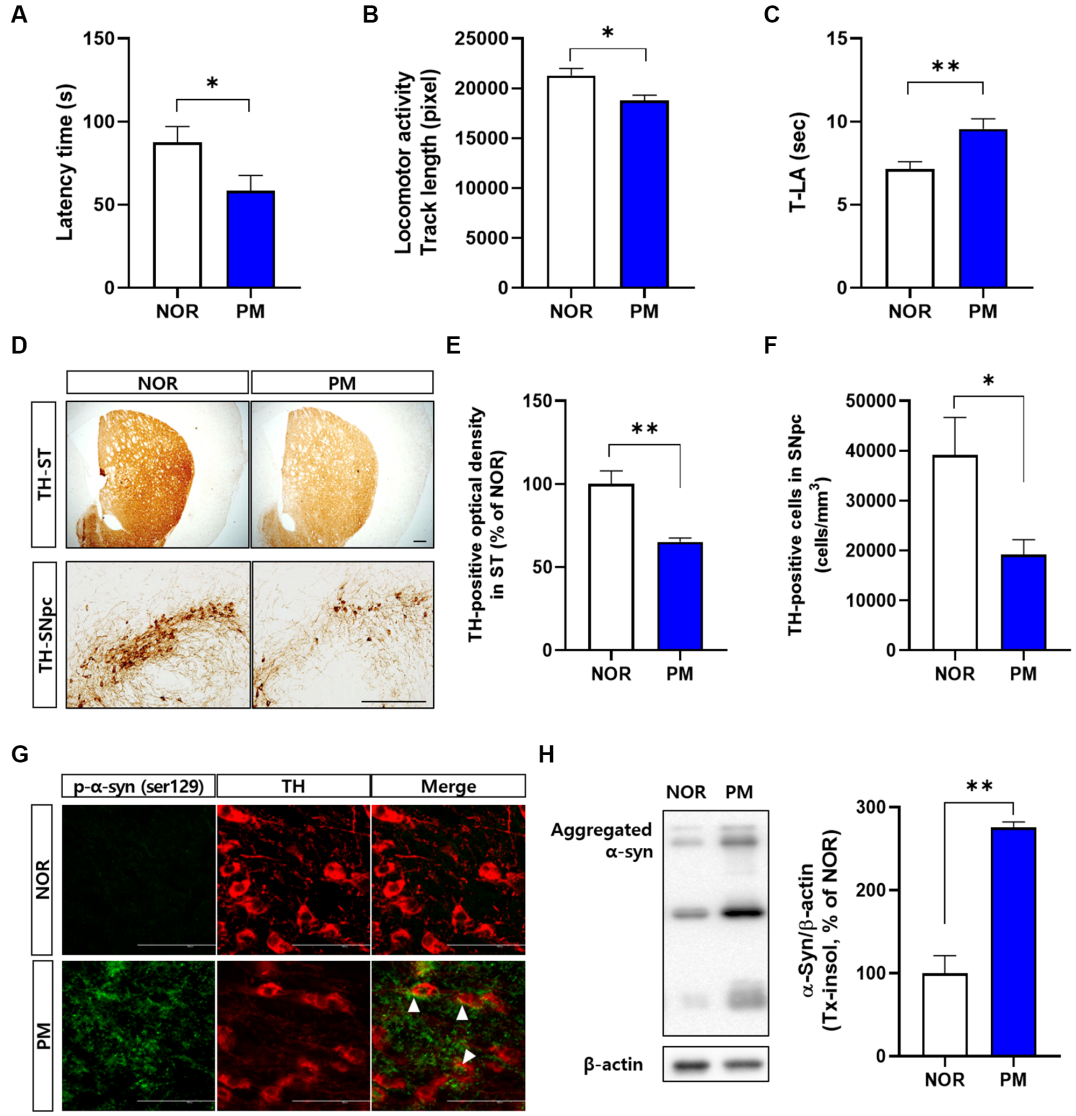
Phenotypes of *P.mirabilis*-induced PD mouse. Graphs of motor functions evaluated by the rotarod test **(A)**, open-field test **(B)**, and pole test **(C)**. Representative photomicrographs **(D)** and graphs of TH-positive fibers in ST **(E)** and TH-positive cells in SNpc **(F)**. Scale bar = 200 μm. Representative photomicrographs **(G)** and western blotting images and graphs of α-syn expression **(H)**. Scale bar = 100 μm. **(A–C)** *N* = 8 per group, **(D–H)** *N* = 4 per group. Values are given as the mean ± SEM. Significant differences were determined by unpaired student’s t-test. *p < 0.05 and **p < 0.01 compared with the normal group. NOR, Normal group, PM, *P.mirabilis* group.

First, we evaluated the severity of behavioral and brain pathological abnormalities of the designed model by *P. mirabilis* in the study. The results showed that motor functions deteriorated in *P. mirabilis*-treated mice compared to those in normal mice. Specifically, we performed three-different experiments to measure motor function. In the rotarod test, latency time of mice treated with *P. mirabilis* (58.43 ± 9.29 s) was significantly shortened than that of mice treated with saline (87.57 ± 9.54 s, Figure 1A). The results of the open-field test showed that track length, which detects traces of the mouse for 30 min, showed that mice treated with *P. mirabilis* (18781.00 ± 551.60 pixels) had uncoordinated movement compared to the Normal group (21272.00 ± 730.90 pixels, Figure 1B). In Figure 1C, we confirmed that the time to landing was significantly delayed in the *P. mirabilis* group (9.55 ± 0.61 s) compared to the Normal group (7.14 ± 0.45 s) in the pole test. In histological analysis, both of dopaminergic neuron terminals and cells were significantly decreased in the brain of *P. mirabilis*-treated mouse (65.23 ± 2.27% of normal in the ST; 19206.00 ± 2963.00 cells in the SNpc) compared to the those of normal mice (100.00 ± 7.98% of normal in the ST; 39161.00 ± 7513.00 cells in the SNpc, Figures 1D–F). In addition, the pathological α-syn aggregation (phosphorylated- or insoluble form) in SN was increased in *P. mirabilis*-treated mouse (insoluble α-syn; 275.5 ± 6.76% of normal in the SN) compared to the that of mice treated with saline (100 ± 21.15% of normal in the SN, Figures 1G,H).

### Metabolic changes in the normal and *Proteus mirabilis*-treated mouse colon

**Figure 2:**
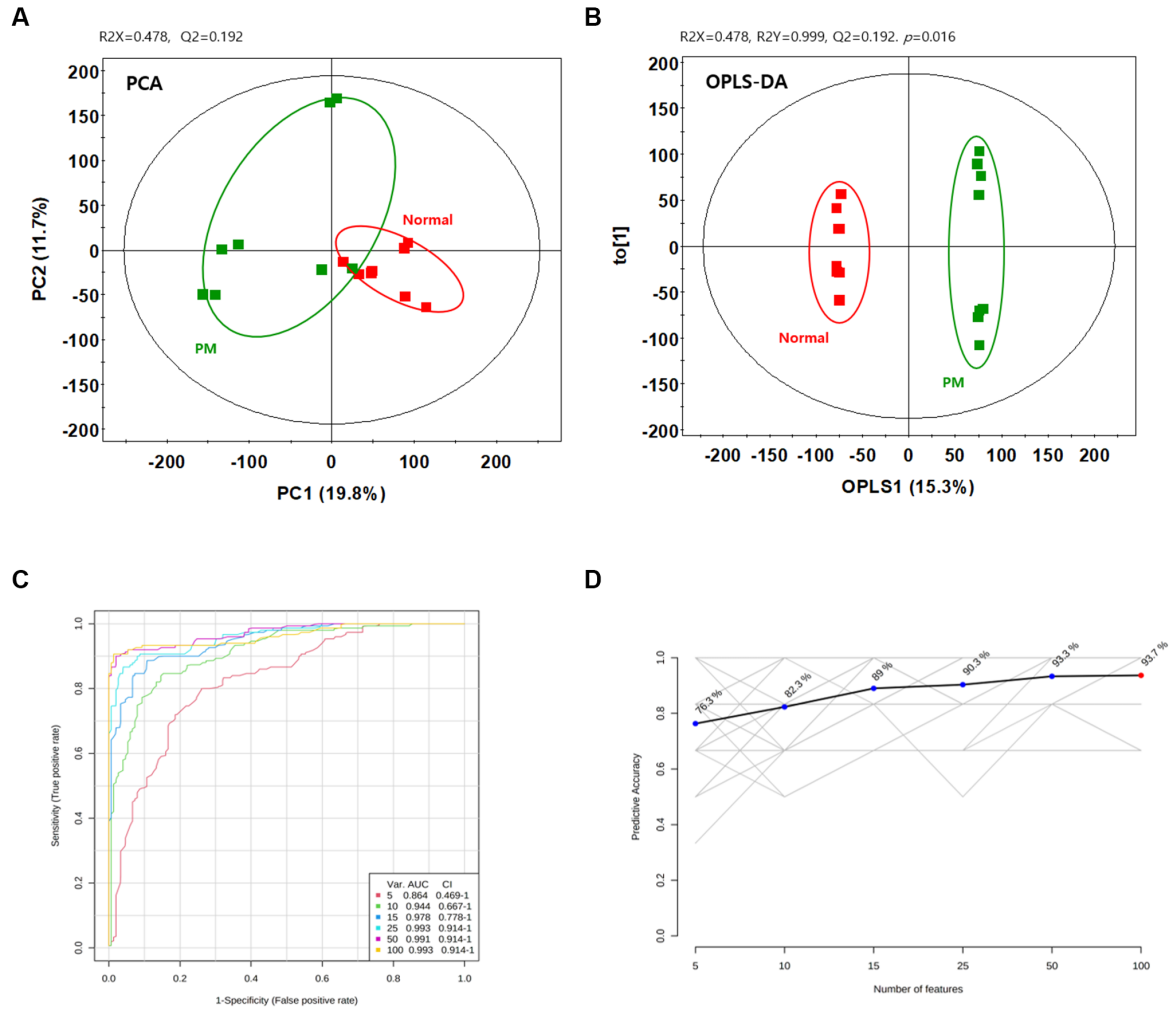
Metabolic changes in the normal and *P.mirabilis*-treated mouse colon. Graphs of PCA **(A)** and OPLS-DA **(B)** between normal and *P.mirabilis*-treated mouse colon. Graphs of ROC curve **(C)** and prediction accuracy **(D)** in multivariate ROC analysis.

To assess the variation between the measured metabolomes in the colon by *P. mirabilis*-induced pathological characteristics, PCA and OPLS-DA were used. Figures 2A,B reported that the groups were clearly discriminated by PCA and OPLS-DA. Also, multivariate ROC analysis was conducted to measure the appropriate degree of variables, and the area under the curve (AUC) and 95% confidence intervals were also measured. The results showed that the prediction accuracy gradually increased, followed by the number of variables (Figures 2C,D). Then, we defined the measured variance in OPLS-DA prediction models, calculated VIP scores, and clarified the tentative metabolites with VIP scores over 0.7 (Table 1).

**Table 1:** Lists of tentative metabolites of altered in *P. mirabilis*-treated mouse colon.

NO.	Tentative metabolites^a^	RT (min)	MS	MS pattern^b^	TMS	Ref.^c^	Fold change^d^	*p*-value
1	Alanine	5.74	116	59, 73, 116, 147, 190, 218	2	STD/MS	0.8	0
2	Valine	6.94	144	73, 75, 100, 144, 218, 246	2	STD/MS	1.11	0.045
3	Serine	8.33	188	73, 100, 147, 188, 204, 218	3	STD/MS	1.15	0.070
4	Aspartic acid	8.88	160	73, 117, 130, 147, 160, 245	3	STD/MS	1.36	0.157
5	Phenylalanine	9.97	146	73, 91, 120, 130, 146, 222	1	STD/MS	1.51	0.099
6	Lysine	12.69	317	73, 128, 156, 174, 230, 317	4	STD/MS	0.45	0.006
7	Tyrosine	12.82	280	73, 100, 147, 179, 218, 280	3	STD/MS	1.07	0.167
8	Lactic acid	5.28	191	66, 73, 117, 147, 191, 219	2	STD/MS	1.02	0.274
9	Methylphosphoric acid	6.57	241	73, 133, 163, 211, 241, 256	2	MS	1.33	0.001
10	Phosporic acid	7.55	299	73, 133, 147, 207, 299, 314	3	STD/MS	0.97	0.181
11	Succinic acid	7.87	147	55, 73, 75, 147, 174, 247	2	STD/MS	0.91	0.000
12	Glyceric acid	8.06	189	73, 103, 133, 147, 189, 205	3	MS	0.67	0.151
13	Aminomalonic acid	9.32	320	73, 133, 147, 174, 218, 320	3	MS	1.74	0.159
14	Malic acid	9.44	233	73, 147, 175, 233, 245, 307	3	STD/MS	1.08	0.090
15	Galacturonic acid	12.94	333	73, 147, 160, 189, 217, 333	5	STD/MS	2.91	0.000
16	N-Indolylacetic acid	14.44	339	73, 96, 117, 129, 145, 339	-	MS	0.79	0.004
17	Glucose	12.61	319	73, 103, 147, 205, 217, 319	5	STD/MS	1.46	0.027
18	Galactose	12.76	319	73, 103, 147, 205, 217, 319	5	STD/MS	1.74	0.017
19	myo-Inositol	13.48	318	73, 147, 191, 217, 305, 318	6	MS	0.88	0.130
20	Palmitoleic acid	13.28	311	73, 96, 117, 129, 145, 311	1	STD/MS	0.7	0.001
21	Myristic acid	12.1	285	73, 117, 129, 132, 145, 285	1	STD/MS	0.58	0.001
22	Palmitic acid	13.39	313	73, 117, 132, 145, 313, 328	1	STD/MS	0.75	0.001
23	Linoleic acid	14.42	337	73, 95, 117, 129, 150, 337	1	STD/MS	0.05	0.067
24	Stearic acid	14.57	341	73, 117, 132, 145, 341, 356	1	STD/MS	0.75	0.000
25	Monostearin	17.41	399	73, 129, 147, 205, 267, 399	2	MS	1.26	0.006
26	Aminoethanol	7.45	100	59, 73, 86, 100, 147, 174	3	MS	0.79	0.000
27	Glycerol	7.51	205	73, 103, 117, 133, 147, 205	3	MS	1.23	0.000
28	Urea	7.22	189	66, 73, 147, 171, 189, 204	2	STD/MS	1.34	0.015
29	Thymine	8.72	255	73, 100, 113, 147, 255, 270	2	STD/MS	1.37	0.007
30	Taurine	10.95	326	73, 100, 147, 174, 188, 326	3	STD/MS	0.82	0.027
31	O-Phosphorylethanolamine	11.77	299	73, 100, 174, 299, 315, 414	4	MS	1.09	0.044
32	Hypoxanthine	11.93	265	73, 125, 193, 206, 265, 280	2	STD/MS	1.22	0.000
33	Xanthine	13.37	353	73, 147, 279, 294, 353, 368	3	MS	1.68	0.009
34	Inosine	16.51	281	73, 103, 147, 217, 230, 281	4	MS	1.49	0.025

a Metabolites identified based on the variable importance projection (VIP) analysis results (with a cut-off value of 0.7) from OPLS-DA.

b MS fragmentation is the fragmentation of the tentative compound.

c MS mass spectrum was consistent with those of NIST and in-house libraries. Standard compound (STD) mass spectrum was consistent with that of the standard compounds.

d Relative levels of metabolites were converted into fold changes between normal and *P.mirabilis*-treated mouse colon. RT, Retention time; TMS, Trimethylsilyl; Ref, references.

### Significant colonic metabolic features which classify *Proteus mirabilis*-induced disease progression

**Figure 3:**
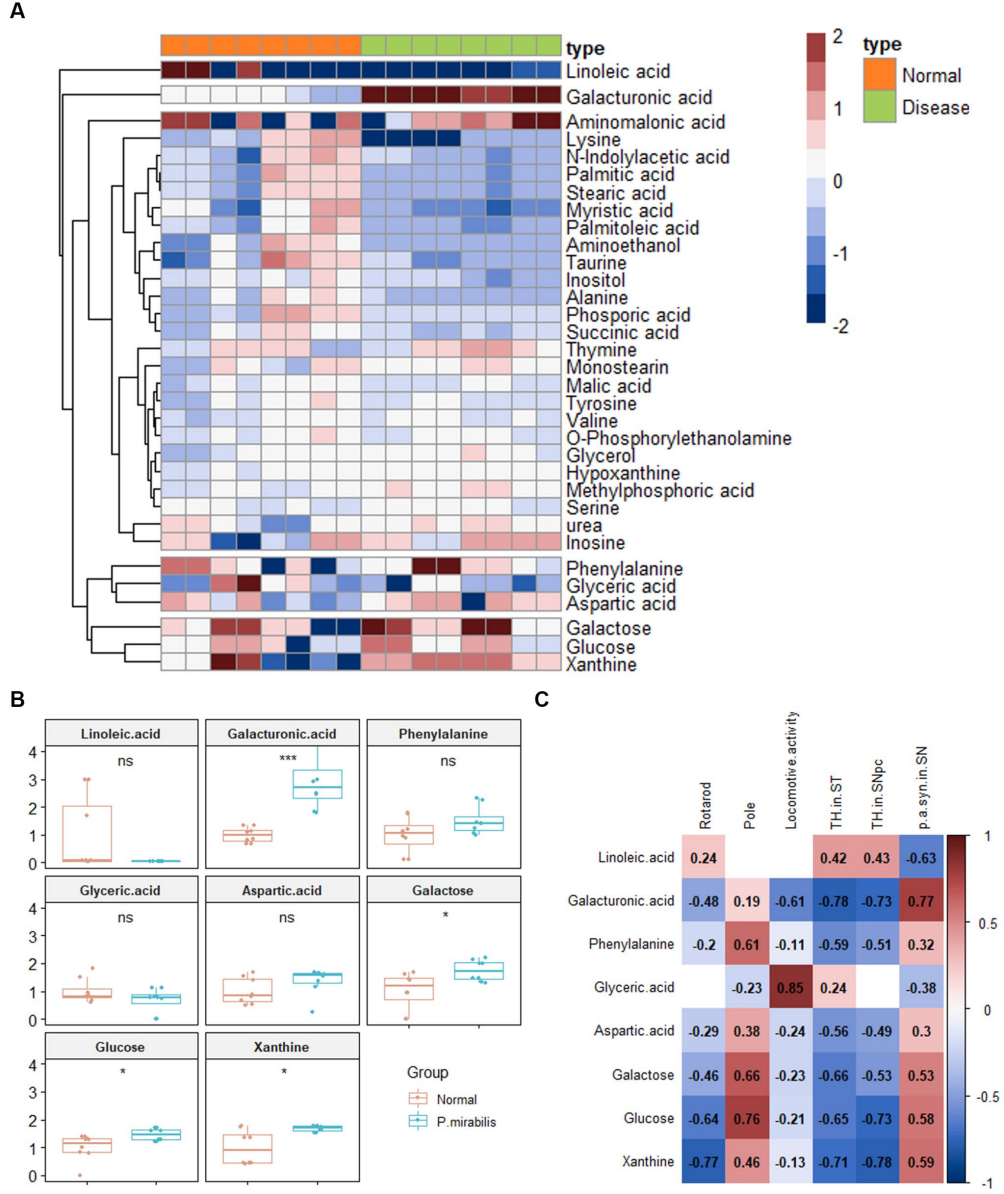
Metabolic alterations in *P.mirabilis*-treated mouse colon. Heatmap for hierarchical pattern analysis **(A)**. Graphs of relative contents of metabolites in colon samples **(B)**. Pearson’s correlation analysis between metabolites and *P.mirabilis*-induced mouse motor dysfunction and brain degeneration **(C)**. Significant differences were determined by unpaired student’s t-test. *p < 0.05 and ***p < 0.001 compared with the normal group.

34 Metabolites were identified in the colon. To investigate the discriminated metabolites between the normal and disease (*P. mirabilis*-treated) status, we conducted a hierarchical pattern analysis (Figure 3A). The hierarchy of colonic metabolites was divided into five clusters: cluster 1, linoleic acid; cluster 2, galacturonic acid; cluster 3, metabolites classified in Amino acids; cluster 4, phenylalanine, glyceric acid and aspartic acid; cluster 5, galactose, glucose, and xanthine. Among them, we chose four differentiated clusters (clusters 1, 2, 4, and 5) to represent PD features. In the selected 8 metabolites, we evaluated the relative quantification of eight selected metabolites in the mouse colon. Figure 3B reports that galacturonic acid, galactose, glucose and xanthine levels were significantly different between normal and disease groups. Pearson correlation coefficients were also calculated for each significant variable analyzed by clustering to investigate the association between phenotypes. Surprisingly, metabolites related to sugar class were strongly associated with an increase in α-syn aggregation, neuronal cell death and motor dysfunctions (Figure 3C).

### Alterations in plasmic metabolites associated with *Proteus mirabilis*-induced motor dysfunctions

**Figure 4:**
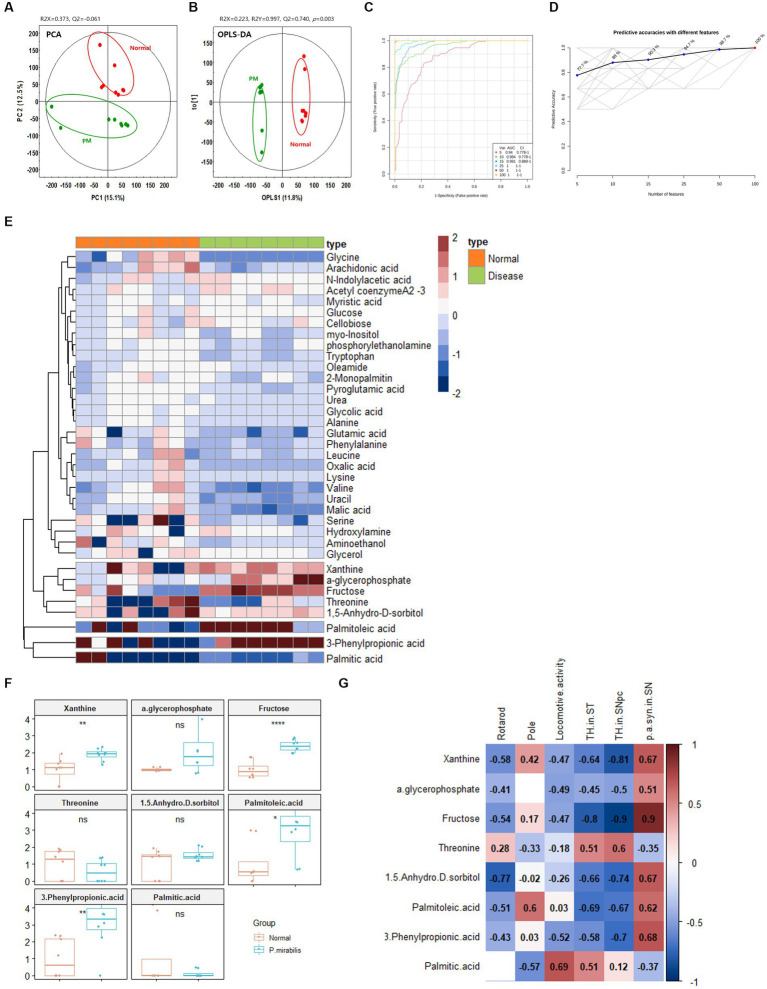
Metabolic alterations in *P.mirabilis*-treated mouse plasma. Graphs of PCA **(A)** and OPLS-DA **(B)** between normal and *P.mirabilis*-treated mouse plasma. Graphs of ROC curve **(C)** and prediction accuracy **(D)** in multivariate ROC analysis. Heatmap for hierarchical pattern analysis **(E)**. Graphs of relative contents of metabolites in plasma samples **(F)**. Pearson’s correlation analysis between metabolites and *P.mirabilis*-induced mouse motor dysfunction and brain degeneration **(G)**. Significant differences were determined by unpaired student’s *t*-test. ^*^p < 0.05, ^**^p < 0.01 and ****p < 0.0001 compared with the normal group.

Based on the above results, we additionally conducted metabolome analysis in mouse plasma to confirm the relationship with colonic metabolites in *P. mirabilis*-treated mouse. According to the colonic results, metabolic proportions were clearly discriminated in the mouse plasma samples by PCA and OPLS-DA (Figures 4A,B). In the plasma samples, we evaluated the AUC and confidence interval using the ROC analysis (Figures 4C,D). Based on the results, we defined the measured variance in OPLS-DA prediction models, and identified 37 tentative metabolites with VIP scores over 0.7 in the plasma (Table 2). To investigate the metabolites discriminated between the normal and disease (*P. mirabilis*-treated) status, we conducted hierarchical pattern analysis (Figure 4E). The hierarchy of cytoplasmic metabolites was divided into five clusters: cluster 1, crude (amino acid etc); cluster 2, xanthine, α-glycerophosphate, fructose, threonine, and 1,5-anhydro-D-sorbitol; cluster 3, palmitoleic acid; cluster 4, 3-phenylpropionic acid; cluster 5, palmitic acid. We than selected eight metabolites as colonic results, evaluated their relative quantification and found that xanthine, fructose, palmitoleic, acid and 3-phenylpropionic acid were significantly associated to disease status (Figure 4F). Pearson correlation coefficients indicated that these metabolites reflected *P. mirabilis*-induced brain neurodegeneration (Figure 4G).

**Table 2:** Lists of tentative metabolites of altered in *P.mirabilis*-treated mouse plasma.

NO.	Tentative metabolites^a^	RT (min)	MS	MS pattern^b^	TMS	Ref.^c^	Fold change^d^	*p* value
1	Alanine	5.74	147	59, 73, 116, 147, 190, 218	2	STD/MS	0.829	0.004
2	Glycine	5.92	102	59, 73, 102, 147, 176, 204	2	STD/MS	0.164	0.000
3	Leucine	6.3	86	75, 86, 103, 146, 170, 188	1	STD/MS	0.66	0.044
4	Valine	6.94	218	73, 75, 100, 144, 218, 246	2	STD/MS	0.272	0.000
5	Threonine	7.72	219	57, 73, 117, 130, 147, 219	2	STD/MS	0.577	0.000
6	Serine	8.33	100	73, 100, 147, 188, 204, 218	3	STD/MS	0.588	0.243
7	Pyroglutamic acid	9.78	156	73, 84, 147, 156, 230, 258	2	MS	0.78	0.007
8	Glutamic acid	10.49	246	73, 128, 147, 156, 246, 348	3	STD/MS	0.434	0.019
9	Phenylalanine	10.61	192	73, 100, 147, 192, 218, 266	2	STD/MS	0.632	0.040
10	Lysine	12.69	317	73, 128, 156, 174, 230, 317	4	STD/MS	0.778	0.039
11	Tryptophan	14.62	202	73, 147, 202, 218, 291, 303	3	STD/MS	0.781	0.093
12	Lactic acid	5.28	219	66, 73, 117, 147, 191, 219	2	STD/MS	0.753	0.000
13	Glycolic acid	5.44	205	66, 73, 133, 147, 177, 205	2	STD/MS	0.804	0.000
14	Oxalic acid	6.05	147	73, 133, 147, 175, 190, 219	2	STD/MS	0.577	0.285
15	3-Phenylpropionic acid	8.85	207	75, 91, 104, 147, 207, 222	1	MS	3.187	0.007
16	Malic acid	9.44	147	73, 133, 147, 175, 233, 245	3	STD/MS	0.248	0.000
17	N-Indolylacetic acid	14.44	339	73, 96, 117, 129, 145, 339	-	MS	1.293	0.003
18	α-glycerophosphate	11.61	357	73, 147, 218, 299, 357, 445	4	MS	2.125	0.037
19	1,5-Anhydro-D-sorbitol	12.27	191	73, 103, 129, 147, 191, 271	4	MS	1.54	0.117
20	Fructose	12.43	307	73, 103, 133, 147, 217, 307	5	STD/MS	2.392	0.000
21	Glucose	13.11	217	73, 103, 147, 191, 204, 217	4	STD/MS	1.208	0.091
22	myo-Inositol	13.48	318	73, 147, 191, 217, 305, 318	6	MS	0.816	0.159
23	Cellobiose	17.5	361	73, 103, 147, 204, 217, 361	8	MS	1.137	0.363
24	Myristic acid	12.1	145	73, 117, 129, 132, 145, 285	1	STD/MS	1.077	0.050
25	Palmitoleic acid	13.28	311	73, 96, 117, 129, 145, 311	1	MS	3.023	0.014
26	Palmitic acid	13.39	117	73, 117, 132, 145, 201, 313	1	STD/MS	0.113	0.200
27	Arachidonic acid	15.34	361	73, 91, 106, 117, 150, 175	1	STD/MS	0.665	0.076
28	Oleamide	15.57	338	75, 116, 131, 144, 198, 338	1	STD/MS	0.936	0.326
29	2-Monopalmitin	16.28	147	73, 103, 129, 191, 218, 313	2	MS	0.87	0.091
30	Hydroxylamine	5.89	59	73, 86, 119, 133, 147, 249	3	MS	1.177	0.273
31	Acetyl coenzymeA2–3	6.17	235	73, 100, 133, 147, 220, 235	-	MS	1.261	0.000
32	Urea	7.22	189	66, 73, 99, 147, 171, 189	2	STD/MS	0.952	0.179
33	Aminoethanol	7.45	174	59, 73, 86, 100, 147, 174	3	MS	0.733	0.243
34	Glycerol	7.51	205	73, 103, 117, 133, 147, 205	3	MS	1.145	0.322
35	Uracil	8.15	241	73, 99, 113, 147, 241, 256	2	STD/MS	0.386	0.000
36	Phosphorylethanolamine	11.77	299	73, 100, 114, 174, 188, 299	5	MS	0.795	0.222
37	Xanthine	13.37	353	73, 147, 279, 294, 353, 368	3	MS	1.876	0.006

a Metabolites identified based on the variable importance projection (VIP) analysis results (with a cut-off value of 0.7) from OPLS-DA.

b MS fragmentation is the fragmentation of the tentative compound.

c MS mass spectrum was consistent with those of NIST and in-house libraries. Standard compound (STD) mass spectrum was consistent with that of the standard compounds.

d Relative levels of metabolites were converted into fold changes between normal and *P.mirabilis*-treated mouse plasma. RT, Retention time; TMS, trimethylsilyl; Ref, References.

### Pathway enrichment analysis in *Proteus mirabilis*-treated mouse

**Figure 5:**
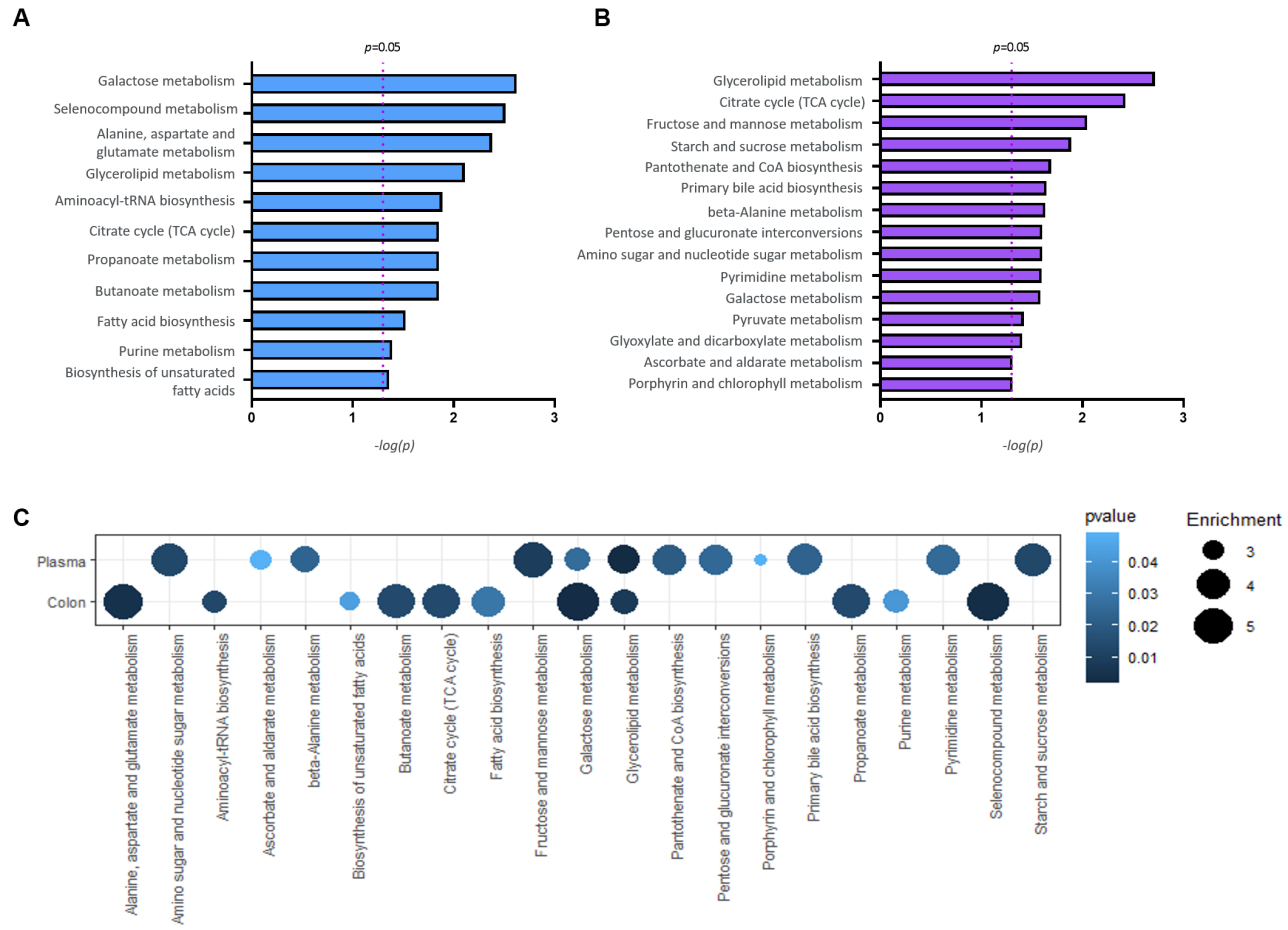
Results of pathway analysis. Bar charts report significant pathways for colon **(A)** and plasma **(B)**. A bubble chart displaying the common significant pathways between samples of normal and *P. mirabilis*-treated mouse **(C)**; the bubble size refers to the enrichment factor of the pathway and the color represents the pathway *p*-value.

Pathway enrichment analysis was performed to explore alterations in metabolic pathways with respect to the disease status. The analysis was performed independently for colon and plasma samples using an unpaired Student’s *t*-test between normal and *P. mirabilis* groups. Figures 5A,B show the significant metabolic pathways in the mouse colon and plasma sample, respectively. In the colon, the results indicated that the metabolic pathway has a high significance in the order of galactose, selenocompound, and alanine, aspartate and glutamate metabolic pathways. In the plasma, glycerolipid, citrate cycle, and fructose and mannose metabolic pathways were ranked in order. We also investigated the enrichment factors associated with the disease. In both the colon and plasma samples, results reveal the galactose and the glycerolipid metabolisms were the most important pathways linked to *P. mirabilis*-treated mice. In addition, we confirmed that fructose and mannose metabolism and pentose metabolism and glucuronate interconversion showed the next highest enrichment factors in the plasma (Figure 5C).

## Discussion

This study presents a metabolomic evaluation of motor dysfunction and neurodegeneration in *P. mirabilis*-treated mice. To understand the influence of altered metabolites on the biochemical impairment, we investigated the associations of peripheral metabolites with *P. mirabilis*-induced disease manifestations. Tentative metabolites identified by GC–MS non-targeted metabolomics were rearranged by clustered pattern analysis to represent the differences between the normal and *P. mirabilis*-treated groups. The canonical metabolites were classified by fatty acids, sugar, amino acids, and TCA acids, which was consistent with the pathway analysis by chemical structure (Supplementary Figure S3). Among them, metabolites related to sugar metabolism were significantly different between the normal and *P. mirabilis*-treated mice in relative quantitation. Moreover, correlation analysis between metabolic alterations and disease manifestations and pathway enrichment analysis indicated that metabolic disturbances in pathways related to sugar metabolism might be involved in *P. mirabilis*-induced pathogenesis.

The microbiota interacts with host metabolism physiologically and pathologically (Agus et al., 2021). Alterations in microbiota-derived or endogenous metabolites are involved in various diseases and disease progression. For example, changes in fecal metabolites of patients with autism have been confirmed to be involved in various metabolic pathways, which induce oxidative stress, neurotransmitter imbalance and mitochondrial dysfunction (Peralta-Marzal et al., 2021). Wu et al. reported a decrease in the levels of microbiota-derived short-chain fatty acids (SCFA), such as propionic acid, butyric acid, and caproic acid, in the blood of PD patients, and among them, propionic acid is correlated with disease progression (Wu et al., 2022). In addition, altered intestinal microbiota regulates tryptophan metabolism by interacting with enterochromaffin cells, resulting in intestinal inflammation (Drobny et al., 2021). The results of this study showed a trend consistent with those of previous studies. In particular, as the decrease in SCFA was confirmed in the same way as in a previous study (Choi et al., 2018), additional studies would be needed regarding the effects of metabolites altered in the model used in this study on CNS-related diseases. In this study, when using a microbiota-mediated brain neurodegeneration model, changes in intestinal microbiota flora and related pathological factors can be major mediating factors. In this regard, it is necessary to consider the correlation analysis with metabolites changed through additional strain analysis and the correlation with intestinal pathology.

The metabolites derived from *P. mirabilis*-treated mouse colon and plasma were confirmed to be involved in energy, lipid, and sugar metabolism. Among them, changes in TCA cycle-related metabolites of *P. mirabilis*-treated mouse colon were notable in relation to energy metabolism. It is closely related to mitochondrial function (Rama Rao and Norenberg, 2012); thus, it is expected to be correlated with enteric neuronal death in the intestine or the leaky gut phenomenon (Urbauer et al., 2020; Smith et al., 2021). Alterations in glycerolipid metabolism identified in colonic metabolites are representative metabolic pathways reported in various neurological disorder models (Gong et al., 2021). These results could help understand the MGB axis-mediated pathological mechanisms and further study on the specific mechanism of *P. mirabilis*-induced neurodegeneration mediated by altered metabolome is needed.

Metabolic analysis studies have been conducted in existing PD patients and various animal models. However, most of the results were measured in cerebrospinal fluid, postmortem brain tissue, etc., and analysis in plasma samples has only been conducted relatively recently. It is known that changes in the tryptophan series were found to be significant in clinical practice, and in addition to this, changes in the metabolome of phenylalanine and energy metabolism-related pathways were prominent (Shao et al., 2021; LeWitt et al., 2023). However, in the case of PD patients, since most of the cases of taking drugs such as levodopa can affect the metabolites, it may not be established whether pathological changes can be reflected (Gonzalez-Riano et al., 2021; Gatarek et al., 2022). In the case of mouse models, although variously known, changes in lipid-based metabolomes and bile acid synthesis-related metabolomes were found to be effective (Graham et al., 2018; Zhang Y. et al., 2021). The difference between the mouse model shown in previous reports and the results of this study needs to be further compared through additional analysis.

Although *P. mirabilis* is known as a major bacteria of urinary tract infection, the role of *P. mirabilis* in relation to intestinal diseases has recently been suggested. Previously, *P. mirabilis* was known to induce inflammatory bowel disease including irritable bowel syndrome and diarrhea (Seo et al., 2015; Gong et al., 2019). In particular, as it was recently revealed that it is closely related to the onset of crohn disease, the importance of *P. mirabilis* in intestinal diseases is increasing (Zhang J. et al., 2021). This action may be related to the pathological changes in the intestines seen in the model used in this study (Choi et al., 2018) (Supplementary Figure S2). From the viewpoint of metabolomics studies, there has been no case of use in a disease model induced by *P. mirabilis*. When metabolomic analysis was performed in other intestinal diseases, slightly different results from those of this study were confirmed. Metabolic changes related to phenylethylamine were prominent in the inflammatory bowel disease mouse model, and branch chain amino acid changes were confirmed in the diarrhea mouse model caused by *Shigella flexneri* (Yu et al., 2018; QS Medeiros et al., 2019). Based on this, it is necessary to additionally compare metabolomes that show significant correlations in *P. mirabilis*-induced intestinal and encephalopathy with metabolites in other intestinal disease models.

Sugar metabolism showed significant changes in *P. mirabilis*-treated mouse colon and plasma. Unlike other metabolic pathways and metabolites, significant changes in sugar metabolism were similarly identified in the colon and plasma; in particular, they had a significant correlation with the brain neurodegeneration. A previous study reported that *P. mirabilis* is the methylglyoxal-producing microbiota, which is the precursor of advanced glycation end-products (AGEs). Its amine oxidase activity contributes to methylglyoxal production from aminoacetone (Baskaran et al., 1989). This suggests that the sugar metabolism plays a crucial role in the pathology of *P. mirabilis*. Sugar metabolites (i.e., glucose, and fructose) are essential for energy metabolism, and those of them, especially for glucose and galactose metabolism, which is closely related to glycerolipid metabolism (Belarbi et al., 2020). Several reports have shown that the metabolic activity of *P. mirabilis* is regulated by central metabolism related to energy metabolism in the human urinary tract (Armitage, 1981; Alteri et al., 2015). Glucose, galactose, and fructose are readily metabolized, generating reducing sugars that covalently react with proteins to generate AGEs that invariably impact protein function (Vicente Miranda and Outeiro, 2010). Among them, protein folding and aggregation have been involved in processes based on those metabolisms and it is also related to microbes known to aggregate proteins (Luna-Pineda et al., 2019; Schramm et al., 2020). For example, *Escherichia coli* is known to form a biofilm with an amyloid protein called curli which induces aggregation of other protein such as α-syn (Sampson et al., 2020). In the case of curli-producing microbes, it is known to act on the metabolic pathway changed in this study (Phue et al., 2005; Ammar et al., 2018), therefore, it could be inferred as their similarities in terms of function. Intriguingly, diabetes, a widespread condition characterized by impaired glucose metabolism, has been established as an important risk factor for PD. AGE levels are increased in the brains of patients with synucleinopathy, and AGEs can be detected at the periphery of Lewy bodies (Sun et al., 2012). This suggests that an excess of carbonyl compounds may play a role in the pathogenesis of PD. Recently, Miranda et al. found that glycation affected primarily the N-terminal region of α-synuclein, reduces membrane binding, impairs α -synuclein clearance, and promotes the accumulation of toxic oligomers that impair neuronal synaptic transmission (Vicente Miranda et al., 2017). In addition, Trezzi et al. reported that mannose, threonic acid, and fructose are the biomarker signatures of early stage PD with high sensitivity and specificity in the human cerebrospinal fluid (Trezzi et al., 2017). These findings show that the results of this study are consistent with previous reports, suggesting that sugar metabolism affects neurodegenerative disease progression and is involved in gut-brain crosstalk.

Metabolites related to sugar metabolism are also correlated with the intestinal pathological phenotypes. In particular, as there are several reports that intestinal barrier dysfunction is aggravated when the amount of glucose is increased or when the state of hyperglycemia is present (Thaiss et al., 2018; Zhang X. et al., 2021), changes in related metabolites may have also affected the action of intestinal pathology (Supplementary Figure S2). Therefore, it is highly likely that it worked in the process of disease inducer rather than byproduct, and by deriving a metabolite closely related to the disease through additional target metabolomics related to sugar metabolism, research will be needed in the future.

In conclusion, we suggest that the possible mechanisms underlying the pathogenesis of brain neurodegeneration induced by *P. mirabilis* might be related to glycation. This study has several limitations: it was performed in a small size and sample region, and poor compared with traditional PD animal (MPTP or 6-OHDA mouse model) or human samples; however, it suggests the possibility brain dopaminergic neurodegeneration based on the MGB axis.

## Data availability statement

The raw data supporting the conclusions of this article will be made available by the authors, without undue reservation.

## Ethics statement

The animal study was reviewed and approved by “Animal Care and Use Guidelines” of Kyung Hee University, Seoul, Korea.

## Author contributions

EH, JC, and MO: conceptualization. EH, JC, ML, YC, and DK: data curation. EH and ML: formal analysis. MO and MP: funding acquisition. EH, ML, and JK: investigation. EH, CL, and MO: methodology. MO: project administration. EH and MO: roles or writing-original draft. IJ, HE, DK, H-JP, and CL: writing-review and editing. All authors contributed to the article and approved the submitted version.

## Funding

This study was supported by Basic Science Research Program through the National Research Foundation of Korea funded by the Ministry of Education (NRF-2018R1D1A1B07048099), grants from the National Research Foundation of Korea funded by the Korean government (2022M3A9B6017813).

## Conflict of interest

MP was employed by MetaCen Therapeutics Inc. R&D Center.

The remaining authors declare that the research was conducted in the absence of any commercial or financial relationships that could be construed as a potential conflict of interest.

## Publisher’s note

All claims expressed in this article are solely those of the authors and do not necessarily represent those of their affiliated organizations, or those of the publisher, the editors and the reviewers. Any product that may be evaluated in this article, or claim that may be made by its manufacturer, is not guaranteed or endorsed by the publisher.

## Footnotes

^1^ http://www.metalign.nl
