# Supplementary material for: Peripheral metabolic alterations associated with pathological manifestations of Parkinson’s disease in gut-brain axis-based mouse model
Source: Front Mol Neurosci. 2023 Aug 10;16:1201073. doi: 10.3389/fnmol.2023.1201073 (PMC10447900; doi:10.3389/fnmol.2023.1201073)
Supplement: Supplementary file 1 [file Data_Sheet_1.docx]

Supplementary data

Materials and Methods

Materials

Paraformaldehyde (PFA), phosphate buffer, phosphate buffer saline, ethylene glycol, glycerol, sucrose, hydrogen peroxide, 3,3'-diaminobenzidine, methanol, methoxyamine hydrochloride, N-methyl-N-(trimethylsilyl) trifluoroacetamide, anti-tyrosine hydroxylase (TH) antibody (AB152) were purchased from Merck Millipore (Burlington, MA, USA). Biotinylated goat anti-rabbit IgG antibody (BA-1000), Goat Anti-Rabbit IgG DyLight 594 (DI-1594) and avidin-biotin complex were purchased from Vector Laboratories (Burlingame, CA, USA). ZO-1 polyclonal antibody (40-2200) was purchased from Invitrogen (Waltham, MA, USA).

Animals and administration

Seven-week-old male C57BL/6J mice used in this study were purchased from Daehan Biolink (Eumseong, Korea). After the adaptation for 7 days, mice were housed in separate cages per group (n = 8 per cage) at an ambient temperature of 23 ± 1 °C and relative humidity 60 ± 10% under a 12 h light/dark cycle and were allowed free access to water and food. All animal studies were performed in accordance with the “Principles of Laboratory Animal Care” (NIH publication number 80–23, revised 1996) and approved by the “Animal Care and Use Guidelines” of Kyung Hee University, Seoul, Korea (approval number: KHUASP(SE)-20-029).

Administration of 1-methyl-4-phenyl-1,2,3,6-tetrahydropyridine (MPTP)

Mice were administered with MPTP hydrochloride (30 mg/kg/day in saline, *i.p.* for 5 days). Vehicles of equal volume (0.25 ml) were given to the normal group. Mice were sacrified at the 7th day after the last MPTP injection.

Immunofluorescences

Mouse colon tissues were fixed by PFA and sectioned. The sections were incubated with primary antibodies against TH and ZO-1(1:1000) for overnight, then treated with secondary antibodies conjugated with Dylight 594 (1:500) for 1 h, and observed using a confocal microscope.

Enrichment pathway analysis

Enrichment pathway analysis using chemical structures was executed using the MetaboAnalyst 5.0. Enrichment Analysis in which the data matrix was normalized by median, and auto-scaling method. Library of super-class chemical structures was used for setting parameter.

Supplementary figures


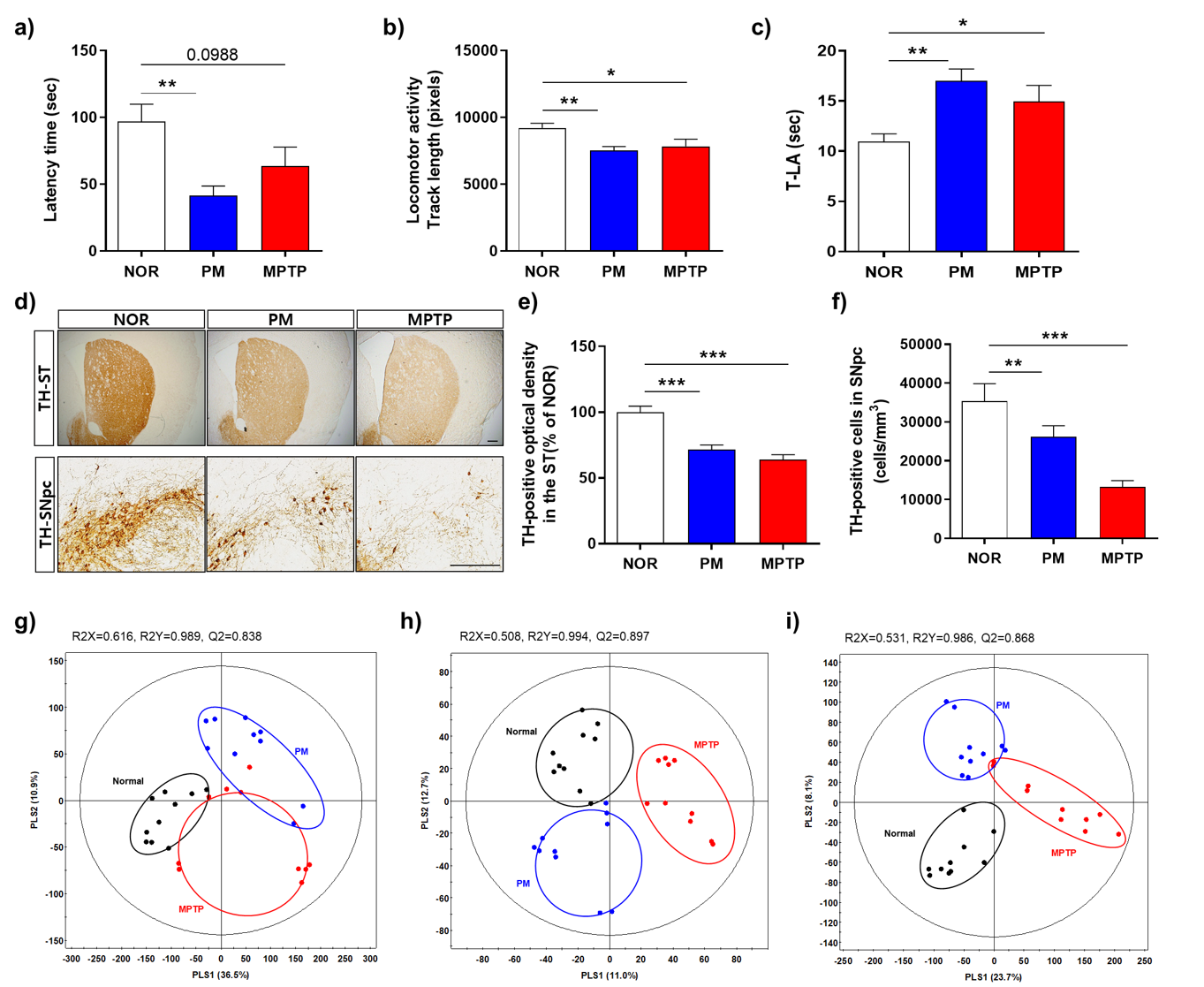


**Fig. S1.** **Phenotypes of MPTP- and *P.mirabilis*-induced PD mouse.** Graphs of motor functions evaluated by the rotarod test a), open-field test b) and pole test c). Representative photomicrographs d) and graphs of TH-positive fibers in ST e) and TH-positive cells in SNpc f). Graphs of PLS-DA among normal, *P.mirabilis*- and MPTP-treated mouse colon g), plasma h) and feces i). Scale bar = 200 μm. Values are given as the mean ± SEM. Significant differences were determined by unpaired student’s t-test. *p < 0.05 and **p < 0.01 compared with the normal group. NOR; Normal group, PM; *P.mirabilis* group.


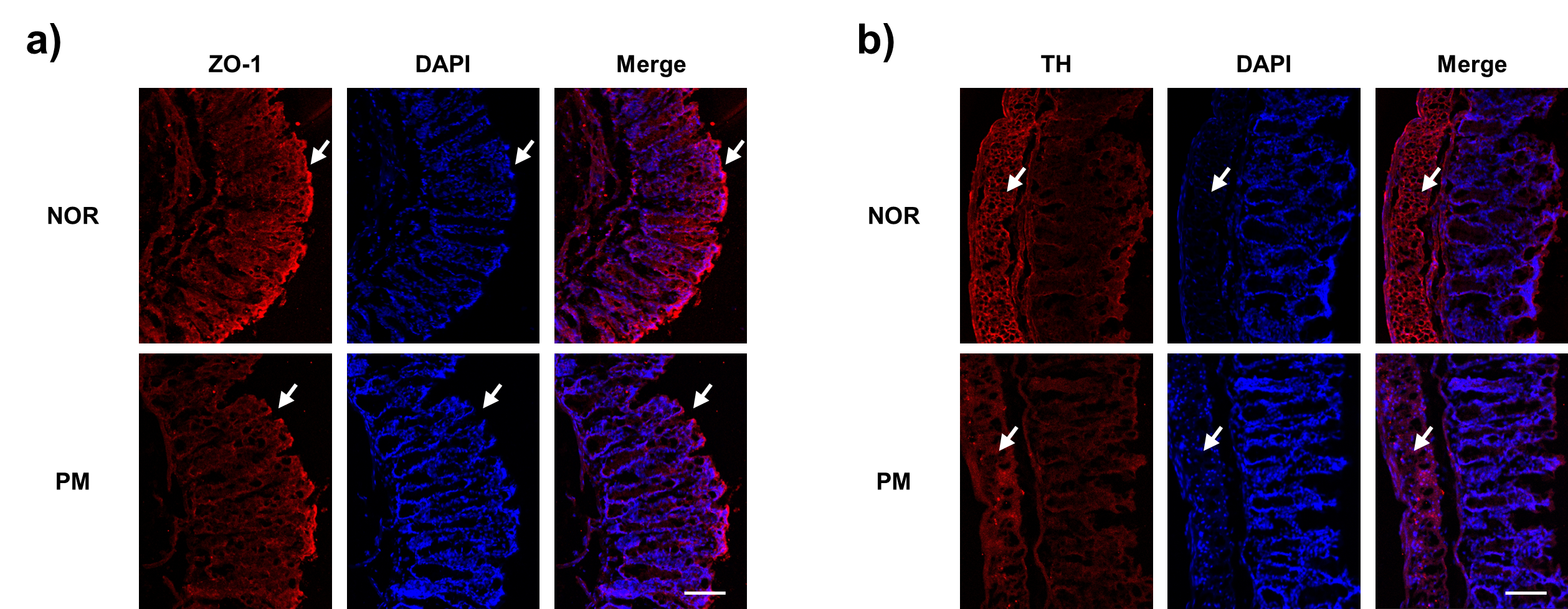


**Fig. S2. Intestinal alterations induced by *P. mirabilis*.** Representative images for ZO-1- a) or TH- b) positive singals in the mouse colon. Scale bar = 100 μm.


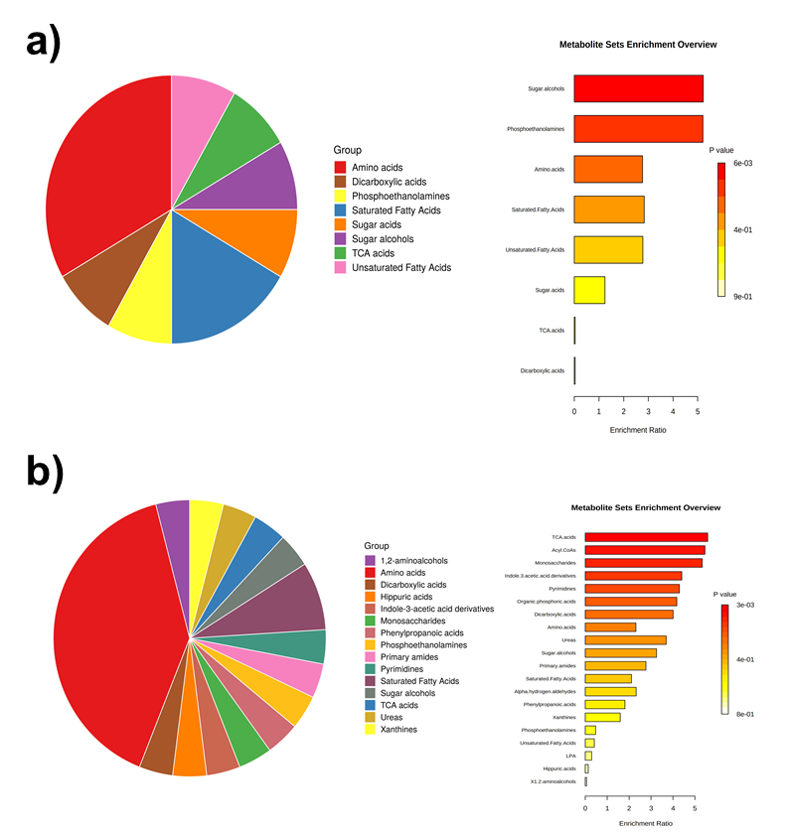


**Fig. S3. Results of pathway enrichment analysis by metabolic category.** Pie (left) and Bar (right) charts report significant metabolic categories for the mouse a) colon and b) plasma.
